# The role of endoscopic ultrasound in the detection of pancreatic lesions in high-risk individuals

**DOI:** 10.1007/s10689-024-00380-5

**Published:** 2024-04-04

**Authors:** Kasper A. Overbeek, Djuna L. Cahen, Marco J. Bruno

**Affiliations:** Erasmus MC Cancer Institute, Department of Gastroenterology & Hepatology, University Medical Center Rotterdam, Rotterdam, The Netherlands

**Keywords:** Endoscopic ultrasound, Endoscopic ultrasonography, High-risk individuals, Pancreatic cancer, Surveillance, Screening

## Abstract

Individuals at high risk of developing pancreatic ductal adenocarcinoma are eligible for surveillance within research programs. These programs employ periodic imaging in the form of magnetic resonance imaging/magnetic resonance cholangiopancreatography or endoscopic ultrasound for the detection of early cancer or high-grade precursor lesions. This narrative review discusses the role of endoscopic ultrasound within these surveillance programs. It details its overall strengths and limitations, yield, burden on patients, and how it compares to magnetic resonance imaging. Finally, recommendations are given when and how to incorporate endoscopic ultrasound in the surveillance of high-risk individuals.

## Introduction

An inherited genetic predisposition is presumed in around 10% of pancreatic ductal adenocarcinoma (PDAC) patients based on familial clustering [1]. In many families, the exact cause of the increased risk remains unidentified, but in around one-third this is explained by an inherited pathogenic variant of one of the known PDAC susceptibility genes, including *LKB1/STK11*, *CDKN2A*, *BRCA1*, *BRCA2*, *PALB2*, *ATM*, *TP53*, and *MLH1/MSH2/MSH6*. Even in the absence of a positive family history, such a pathogenic variant can be found in 3% of PDAC patients [2].

Surveillance of these so-called high-risk individuals is aimed at detecting neoplastic pancreatic lesions at the earliest possible stage: as a stage I cancer that is still limited to the pancreas or, preferably, as a high-grade precursor lesion which includes pancreatic intraepithelial neoplasia 3 (PanIN-3) lesions and intraductal papillary mucinous neoplasms (IPMNs) with high-grade dysplasia. To date, surveillance relies solely on imaging, as available biomarkers are insufficiently able to differentiate between low-grade and high-grade dysplasia, or between neoplastic and non-neoplastic abnormalities.

Imaging techniques unsuited for surveillance include computed tomography (CT), 18-fluorodeoxyglucose positron emission tomography (FDG-PET), and abdominal ultrasound. CT has a relatively low sensitivity for detecting subcentimeter lesions, and the cumulative radiation exposure renders it unfit for repeated imaging [3]. It is, however, still the modality of choice for staging of pancreatic cancers [4–7]. FDG-PET is useful for differentiating pancreatic cancer from mass-forming chronic pancreatitis and for detecting distant metastases [8, 9]. However, its diagnostic performance in detecting small lesions is debatable [10, 11]. Abdominal ultrasound has a low sensitivity for the detection of small pancreatic abnormalities and often visualizes the pancreas incompletely or unclearly, especially in individuals with a high body mass index, making it unsuitable for surveillance [4].

Better suited imaging modalities for surveillance are endoscopic ultrasound (EUS) and magnetic resonance imaging/magnetic resonance cholangiopancreatography (MRI/MRCP). The first attempts of surveillance in high-risk individuals employed EUS as the primary test, with additional endoscopic retrograde cholangiopancreatography (ERCP) and CT in case EUS detected abnormalities [12–16]. From 2009 onwards, surveillance programs started to incorporate MRI/MRCP as a primary test; either alternating or combining it with EUS. Since then, these prospective programs have demonstrated that both can detect high-grade precursor lesions and early cancers during surveillance of high-risk individuals [17–20].

It is still a matter of debate which imaging modality has the best performance to detect pancreatic lesions at the desired early stage. A high diagnostic accuracy is crucial as undertreatment quickly results in advanced cancer given the rapidly progressive nature of PDAC, while overtreatment of low-grade precursors or benign lesions poses high risks of morbidity and mortality associated with pancreatic surgery [21]. In the latest international Cancer of the Pancreas Surveillance (CAPS) consortium consensus guidelines, experts could not reach consensus on which imaging modality is superior [22]. Their conclusion was that both are suitable and surveillance can be performed using either one or both; a conclusion that has been adopted by guidelines of the ACG, ASGE, AGA, and ESGE [23–26].

The aim of the current review is to discuss the strengths and limitations of EUS in detecting pancreatic abnormalities during surveillance of high-risk individuals, such as the diagnostic yield and burden to patients. These characteristics will be compared to those of MRI/MRCP, and areas of insufficient knowledge will be identified. Finally, we give our recommendations when and how to implement EUS in a surveillance program.

## Strengths

### Diagnostic performance

The foremost strength of EUS is its ability to depict the pancreas from close proximity, resulting in optimal detection of (small) pancreatic abnormalities. Multiple studies have reported a high diagnostic accuracy for PDAC detection for EUS, with a sensitivity ranging between 81 and 100% and specificity ranging from 73 to 100%. It outperforms CT, which has a lower sensitivity (53–74%) and slightly lower specificity (53–94%) [27–29]. MRI has the same reported performance as CT [4]. For pancreatic lesions smaller than 3 cm, this superiority over CT and MRI is even clearer [29, 30]. An additional strength of EUS is its high sensitivity in detecting pancreatic neuroendocrine tumors of 80–92%, compared to 63% for CT and 66% for MRI [31, 32], although this is not the main objective of surveillance [22].

EUS may have difficulty in differentiating between neoplastic lesions and other hypoechoic abnormalities due to inflammatory masses, signs of previous inflammation due to chronic or acute pancreatitis, lipomatous parenchyma, or atrophy due to old age. In such cases, the diagnostic accuracy can be improved by performing additional contrast-enhancement EUS [33]. In a study in 277 patients with solid pancreatic lesions, the overall sensitivity and specificity of contrast-enhanced harmonic EUS was 95% and 89% for ductal adenocarcinomas and 79% and 99% for neuroendocrine tumors. In the subgroup of adenocarcinomas smaller than 2 cm, contrast-enhanced EUS outperformed CT, with a sensitivity of 91% and specificity of 94% [34]. This bears important clinical relevance, because the majority of small asymptomatic solid pancreatic lesions are not PDAC in both low-risk and high-risk populations [20, 35], and accurate stratification is essential to prevent unnecessary surgery. In the future, developments in artificial intelligence applied to EUS imaging may prove an additional means to improve its diagnostic accuracy.

### Tissue acquisition

A significant advantage of EUS over MRI/MRCP is the possibility of tissue acquisition through fine-needle aspiration (FNA) or biopsy (FNB). As mentioned, the majority of small asymptomatic solid pancreatic lesions are not PDAC, and a histologic confirmation is a prerequisite before performing an oncological pancreatic resection. Multiple meta-analyses have demonstrated a high diagnostic accuracy of FNA to diagnose PDAC, with a pooled sensitivity ranging between 87% and 91%, and specificity of 94–96% [36, 37]. A more recent meta-analysis reported on the results of 18 randomized controlled trials comparing FNA to FNB. Both were shown to have a good diagnostic accuracy, ranging 78–83% for FNA, and 83–87% for FNB, with a small but significant advantage of FNB.

EUS-FNA may also detect PDACs that are not visible on other imaging modalities. In a retrospective study of 116 patients with clinical findings suggestive of PDAC (based on presentation, laboratory abnormalities, ductal dilation) but no mass on CT-scan, EUS identified a focal mass in 84 patients, of which 44 were a pancreatic ductal adenocarcinoma [38]. In this group, EUS-FNA had a diagnostic accuracy of 91%. In addition, there were 32 patients without a focal mass on CT and EUS, in whom EUS-FNA performed at a narrowing of the bile duct or pancreatic duct revealed six additional cancers. This particular observation deserves attention in the context of surveillance, as PDAC cases in high-risk individuals have been described in which a pancreatic duct dilation was the only preceding sign of malignancy [20, 39].

### Pancreatic juice collection

An added utility of EUS is the possibility to collect pancreatic juice from within the duodenum after stimulation of its excretion by intravenous secretin injection. Pancreatic juice might be a valuable biomarker source as it is secreted directly by the ductal cells from which neoplasia originates, potentially containing more accurate markers than other biomaterials such as serum or feces. This may be beneficial specifically for high-risk individuals, as they already undergo repeated EUS in the course of surveillance. Multiple biomarkers in pancreatic juice, including protein biomarkers like S100P, microRNAs, and cell-free DNA mutations like KRAS, SMAD4 and TP53, are associated with PDAC [40–48]. If proven sufficiently predictive, such markers may provide earlier detection of neoplasia and allow for personalization, such as a tailored surveillance interval or more accurate selection for surgery. In addition, they may be able to differentiate between advanced neoplasia (high-grade dysplasia or PDAC) and low-grade precursor lesions [49], an area in which current imaging-based surveillance lacks [20, 39], but this remains to be determined.

## Limitations

### Interobserver variability

The biggest limitation of EUS is its operator-dependency. It takes relatively long to develop the core skills required to perform EUS [50]. When surveilling high-risk individuals, in which the smallest and earliest signs of potential neoplastic progression are of importance, even more extensive expertise is required. Even among experts, interobserver agreement of EUS images of high-risk individuals has been shown to be poor [51]. Additionally, studies have reported on individuals being selected for surgery because of a suspicious lesion after which histologic evaluation revealed mere low-grade dysplasia or even non-neoplastic lesions, although this is equally true for MRI [39]. There also might be a learning curve of the collective multidisciplinary team involved in a pancreatic cancer surveillance program. In the CAPS study, one of the largest ongoing prospective surveillance studies worldwide, patient selection for surgery improved over time, with less patients resected for low-grade dysplastic lesions [17]. These challenges have resulted in the international CAPS consortium to recommend surveillance only within dedicated multidisciplinary research programs in which extensive expertise can be built up, and that EUS should be used as a primary modality only if executed by expert endosonographers [22].

### Longitudinal comparison

An advantage of MRI/MRCP over EUS is the easier comparison of the findings to previous surveillance MRI/MRCPs. Although EUS images and videos can be stored for future reference, these are usually not accessible during the investigation. In addition, a direct comparison is often difficult, for example due to differences in scope positioning and signal quality. For cystic lesions especially, a direct comparison between visits is useful for determining growth. Fast cyst growth is a worrisome feature for developing malignancy in neoplastic cystic lesions within the general population [52], and has also been shown to be predictive of PDAC in high-risk individuals [17, 20]. Pancreatic cysts may even grow faster in high-risk individuals compared to the general population [53]. Although these findings need further confirmation, they put emphasis on a detailed assessment of size and growth of each cyst at each surveillance visit, and place MRI in a pole position in this respect.

### Complication risk

Diagnostic non-interventional EUS has a very low risk of complications of less than 0.3% [54]. Potential complications include perforations of the esophagus or duodenum, aspiration, or bacteremia. Complications are more likely following FNA or FNB (< 3%), and most often include post-procedural pain, acute pancreatitis, hemorrhage, or fever and infectious complications [55–57]. The procedure-related mortality risk is estimated to be very low, at 0.02–0.19% [55, 56].

Needle-tract seeding after FNA/FNB of a pancreatic malignancy is a potential risk but rare [58]. This mainly concerns seeding into the gastric wall after a biopsy of the body or tail, as tumors in the head of the pancreas are usually biopsied through the duodenal wall, which is subsequently resected in case of malignancy. The possibility of needle-tract seeding has been substantiated by an increasing number of case reports, with a recent systematic review identifying 46 such published cases [59]. A large retrospective Japanese study, not yet published at the time of the systematic review, reported on 9300 patients with resected PDAC who had preoperatively undergone FNA or FNB, and found needle-tract seeding lesions after 0 of 4746 (0%) transduodenal tissue acquisitions, but after 38 of 4435 (0.86%) transgastric tissue acquisitions (*P*-value < 0.001), almost all (97.4%) of which were located in the gastric wall [60]. While relevant, it should be noted that this risk is still likely much lower compared to that of percutaneous biopsies [61]. In terms of clinical outcomes, multiple studies have shown no overall difference in survival or recurrence-free survival between PDAC patients who underwent pre-operative FNA or not [58, 62]. Within the group of patients in whom needle-tract seeding had taken place, the Japanese study did find longer survival (median 52 months versus 26 months) for patients who underwent resection of the needle-tract lesion compared to those who didn’t [60].

### Patient burden

Multiple prospective programs have assessed the psychological impact of imaging-based surveillance on high-risk individuals [63–72]. Although study endpoints and used psychological instruments vary across study designs, overall, they report positive psychological outcomes, such as low degrees of cancer worries, anxiety, depression, and general distress [73], with participants having a lower perceived cancer risk than non-participating high-risk individuals [74]. Studies that evaluated long-term psychological outcomes all reported a decrease in cancer-related distress during surveillance [66, 69, 71, 74, 75].

A few studies assessed differences between the burden of EUS and MRI, and evaluated participants’ preferences. The only head-to-head comparisons come from the Dutch familial pancreatic cancer surveillance program, in which high-risk individuals underwent both EUS and MRI at each visit. In the first evaluation of 66 high-risk individuals, only 10% reported the procedures to be burdensome, without differences between EUS and MRI [63]. In a later study of 140 individuals, results were similar: 11% experienced EUS as burdensome, and 10% MRI. Of note, before their baseline visit, 34% had dreaded their first EUS compared to only 3% their MRI, but this dropped significantly after subsequent follow-up visits to equal levels as MRI (from 9% after the first EUS to 6% after the fourth EUS, and from 8% after the first MRI to 0% after the fourth MRI) [74]. When analyzing a subgroup of participants who had to undergo additional examinations after shortened intervals because of lesions of unknown relevance, or who underwent surgery, more participants preferred EUS over MRI than vice versa [70]. In addition to the results of this study, other EUS-based programs have reported an improvement in psychological outcomes at long-term follow-up, and that receiving a negative EUS or EUS-FNA test result improved quality of life [72].

## Performance within surveillance of high-risk individuals

### Chronic pancreatitis findings

In high-risk individuals, a high prevalence of findings similar to those found in early chronic pancreatitis, such as hyperechoic parenchymal foci, calcifications, hyperechoic pancreatic duct walls, or lobularity, has been reported since the earliest EUS-based surveillance programs were initiated [12, 15, 76, 77]. It is thought that these findings are associated with lobulocentric atrophy caused by PanIN lesions [12, 78], and hence, that their detection could be helpful in identifying patients with neoplastic progression. However, these features did not evolve in one study during a three-year follow-up [79], and patients who underwent resection for such features mostly harbored low-grade PanINs, which are common incidental findings with a low risk of neoplastic progression [16, 80, 81]. If having multiple EUS features of chronic pancreatitis is associated with an increased risk of PanIN-3 lesions, representing the clinically more relevant high-grade dysplasia, remains inconclusive [81].

### Pancreatic cystic lesions

Pancreatic cystic lesions are already highly prevalent in the general population and, although direct comparisons have not been made, their prevalence seems to be even higher among high-risk individuals, including many IPMNs [17, 20, 53, 82–86]. The sensitivity of EUS to detect cystic lesions in high-risk individuals has been compared in a blinded fashion in one study by Harinck et al. They compared baseline EUS to baseline MRI/MRCP in 139 high-risk individuals, and found a sensitivity for cystic lesions of 39% for EUS and 90% for MRI [87]. In the same cohort, the diagnostic yield was revaluated (non-blinded) in 366 individuals after a mean follow-up of 63 months. Again, MRI/MRCP was more sensitive than EUS (83% versus 42%), but this difference was much smaller for cystic lesions ≥ 10 mm (92% versus 70%), and not different for main pancreatic duct dilation (60% versus 62%). Moreover, when looking specifically at high-risk features such as a solid component or mural nodule, EUS outperformed MRI/MRCP (100% versus 20%), although the number of cases was small [20]. Other surveillance studies have also reported detection rates for EUS and MRI, but none performed both tests at the same time during each visit, hampering the comparability of the results.

Similar to early chronic pancreatitis findings, the clinical relevance of the detection of IPMNs in high-risk individuals is unclear. Almost all IPMNs detected in high-risk individuals concern branch-duct IPMNs, which, in the general population, have a low risk of malignancy, and this risk is even lower if they remain stable during several years of follow-up [88, 89]. Also for high-risk individuals, IPMNs were initially deemed of little importance, because their hereditary risk was thought to be the result of a solid precursor pathway rather than a cystic one, based on pathological and genetical analyses of resected familial pancreatic cancers [90, 91]. However, in clinical studies, almost half of neoplastic progressors seem to have arisen from a cystic lesion [39], and fast cyst growth was shown to be a predictor for the development of PDAC in multiple surveillance cohorts [17, 20]. The actual malignancy risk of branch-duct IPMNs in high-risk individuals has not been established, and it remains uncertain if this is higher than in the general population. One study directly compared the growth rate and malignancy risk of IPMNs in 81 high-risk individuals to those in 442 individuals without familial risk, and found that IPMNs in high-risk individuals grew faster and were more likely to develop worrisome growth speeds (≥ 2.5 mm/year) [53]. PDAC risk seemed higher in pathogenic variant carriers (11%) compared to pathogenic-variant negative familial pancreatic cancer kindreds (0%) and individuals without familial risk (1%), although numbers were insufficient to demonstrate statistical significance. The malignancy risk of an IPMN in a pathogenic variant carrier was 23% in case of a growth rate of ≥ 2.5 mm/year, 30% for ≥ 5 mm/year and 60% for ≥ 10 mm/year, warranting more intensive surveillance or even surgical resection in selected cases. The international CAPS consortium consensus guidelines provide guidance on the management of cysts with worrisome features in high-risk individuals [22], which are largely based on the guidelines for sporadic IPMNs in the general population. However, correct selection for surgical resection of IPMNs in high-risk individuals remains challenging, as these selection criteria have been shown to require further scrutiny [92].

### Advanced neoplastic lesions

While the relevance of presumed low-risk abnormalities like branch-duct IPMNs and chronic pancreatitis findings is still unclear, the most important question remains: which imaging test is best suited for reaching the primary goal of surveillance, namely the detection of high-grade dysplasia or PDAC confined to the pancreas? Table 1 lists all prospective surveillance programs that incorporate EUS and their rates of detection for neoplastic progressors overall, and early-stage neoplastic progressors in particular. The difficulty in drawing firm conclusions is that the number of cases is low in all studies, varying from zero to 36, and that almost no programs perform both EUS and MRI/MRCP at the same time. Thus, it cannot be established if EUS or MRI/MRCP is superior in detecting neoplastic progressors.

Three meta-analyses have attempted to combine these results. In 2015, Lu et al. analyzed nine surveillance cohorts and reported that EUS detected 64% of pancreatic cancers versus 43% for MRI, without specifying the detection rates for high-grade dysplasia or early PDAC [93]. In 2018, Signoretti et al. included 16 studies with 1588 high-risk individuals, in which a pooled prevalence of early-stage neoplastic progressors of 3.3% was found [94]. EUS detected more solid pancreatic lesions compared to MRI (5.2% versus 4.1%), but the pooled prevalence of pancreatic lesions considered a successful target of surveillance was similar (2.9% for EUS and 2.5% for MRI). In 2019, Corral et al. analyzed 19 studies comprising 1660 high-risk individuals, with an overall diagnostic yield of high-grade dysplasia or PDAC of 0.74 per 100 patient-years [77]. In this analysis, EUS detected more of such lesions (1.07 per 100 patient-years) than did MRI (0.41), but this difference did not reach statistical significance.

**Fig. 1:**
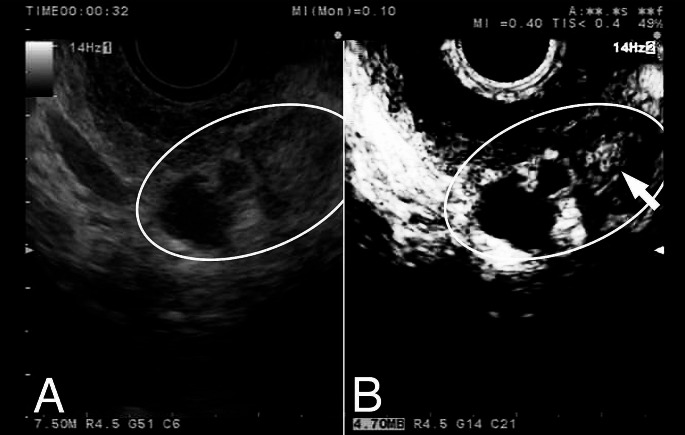
Endoscopic ultrasound images of a branch-duct intraductal papillary mucinous neoplasm (A) detected in the pancreatic tail of a 50-year old *CDKN2A* pathogenic variant carrier. It had grown from 15 to 23 mm in one year and developed a solid component that seemed hypovascular after contrast-enhancement (B). The lesion was visible on MRI/MRCP but not the solid component. After resection, histology revealed a T1cN1M0 intraductal papillary mucinous neoplasm-associated pancreatic ductal adenocarcinoma

**Fig. 2:**
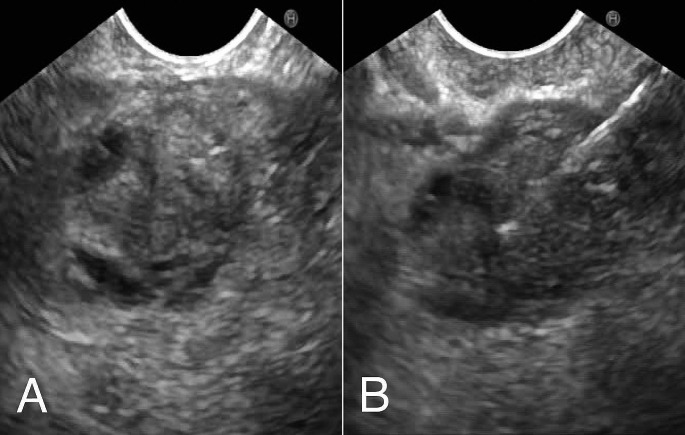
Endoscopic ultrasound images of a 15 mm solid lesion (A) detected in the pancreatic head of a 54-year old patient with Peutz-Jeghers syndrome. The lesion had low uptake of contrast (not shown). On MRI/MRCP the lesion was dubiously present and could not be characterized. On CT it was not visible. At revaluation with endoscopic ultrasound two months later, the lesion was unchanged and fine-needle aspiration (B) was suggestive of malignancy. The lesion was resected and staged as a T1aN0M0 pancreatic ductal adenocarcinoma

While there is not yet convincing evidence that either test is superior, a trend is observed that EUS detects more solid lesions, lesions with high-grade dysplasia and PDAC. This is in line with our experiences within the Dutch familial pancreatic cancer surveillance study, in which EUS detected two early-stage PDACs that were not recognized on MRI/MRCP [20]. Several such cases have also been reported in the CAPS cohort of the Johns Hopkins hospital [3]. In addition, in our cohort we also found two cases in which both MRI/MRCP and EUS detected a cystic abnormality, but only EUS detected the high-risk features that led to surgery, revealing a T1 PDAC in both cases [20]. Figures 1 and 2 show EUS images illustrating these two latter cases. The superior ability of EUS to detect worrisome features and high-risk stigmata has been shown on a wider scale in a multicenter retrospective study by Tamburrino et al. They retrospectively analyzed 837 patients from the general population (without specific increased hereditary risk) undergoing surveillance of branch-duct IPMNs, and identified that EUS-based surveillance was independently associated with a higher detection rate of worrisome features and high-risk stigmata than MRI-based surveillance (HR 2.46, 95% CI 1.74–3.47), although the possibility of selection bias could not be excluded in this study [95].

## Cost-effectiveness

Whether surveillance is cost-effective in high-risk individuals depends on the detection rate of high-grade dysplasia and PDAC and, therefore, on the degree of hereditary risk, which varies greatly between the different subgroups. A recent study estimated that for pancreatic cancer surveillance to be cost-effective, the lifetime PDAC risk needs to be at least 10% [96]. This highest-risk group includes carriers of a pathogenic *CDKN2A* variant and Peutz-Jeghers syndrome patients (lifetime risks estimated at 19% and 11–36%). For the other subgroups, including carriers of lower-risk pathogenic variants such as *BRCA1*, *BRCA2*, *PALB2* and *ATM*, and pathogenic variant-negative familial pancreatic cancer kindreds, it is doubtful if their risk surpasses this threshold [20].

One meta-analysis from the USA compared the costs of EUS to MRI, and found that MRI was the most cost-effective strategy in high-risk individuals with a relative risk five to twenty times that of the general population, but in individuals with a relative risk of twenty or more, EUS was more cost-effective [97]. It should be noted that these results cannot be extrapolated to health care systems outside the USA.

## Conclusions and recommendations

In summary, EUS is a test with a high diagnostic accuracy for detecting pancreatic abnormalities, and is the superior test specifically for lesions smaller than 3 cm. Additional advantages are the improved differentiation of lesions by the application of contrast enhancement, the possibility for tissue acquisition through FNA or FNB with high sensitivity and specificity, and the option to collect pancreatic juice for biomarker analysis. Complication rates are very low, especially for diagnostic EUS without tissue acquisition, and participants experience the burden of surveillance by EUS to be equally low as by MRI. EUS is inferior to MRI/MRCP in detecting any size pancreatic cysts, but this is not the case for cystic lesions larger than one centimeter or for pancreatic duct dilation, and it seems to outperform MRI/MRCP in detecting high-risk features such as solid components or nodules within cysts. While there is no hard evidence that one test has a better diagnostic accuracy for the primary targets of surveillance (high-grade dysplastic precursor lesions and early PDAC), currently available data show a trend favoring EUS. Based on these considerations it was decided in the Dutch familial pancreatic cancer surveillance study to stop performing both tests and to perform EUS as our standard diagnostic imaging modality, with MRI/MRCP performed only at the baseline visit and on indication during follow-up.

EUS is operator-dependent and has a relatively long learning curve that is even greater when used in the context of pancreatic cancer surveillance. Therefore, we recommend to use EUS as a primary surveillance modality only when performed by expert endosonographers, and within a dedicated research program which allows for the accumulation of experience. If these conditions are not met, then MRI/MRCP is a good alternative as a surveillance test. It needs to be stressed that to date, there is only ample evidence that pancreatic cancer surveillance leads to a true survival benefit. Only in pathogenic *CDKN2A* variant carriers, who are among the highest-risk group, has such data become available [98]. For all other risk groups, this remains to be proven and, therefore, surveillance is recommended to be performed only within research programs.

**Table 1:** Detection of neoplastic progressors in prospective surveillance programs of high-risk individuals using endoscopic ultrasound

City, country	Last author, year of publication	Imaging modality (if abnormal findings)	N	Mean follow-up (range, months)	Pathologically-confirmed neoplastic progressors (HGD or PDAC)						
					Total cases (N)	PDAC cases (n)	HGD cases (n)	Detected by EUS in any stage (n/N, %)	Detected by EUS as HGD or early-stage PDAC (n/N, %)	Detected by MRI in any stage (n/N, %)	Detected by MRI as HGD or early-stage PDAC (n/N %)
Seattle, USA	Brentnall, 1999 [12]	EUS (ERCP)	14	0	NR	0	NR	NR	NR	NA	NA
	Rulyak, 2001 [13]	EUS (ERCP)	35	NR (1–48)	NR	0	NR	NR	NR	NA	NA
	Kimmey, 2002 [14]	EUS (ERCP)	46	NR (NR)	NR	0	NR	NR	NR	NA	NA
Baltimore, USA	Canto, 2004 [15]	EUS (ERCP, CT)	38	22 (11–51)	1	1	0	1/1, 100%	0/1, 0%	NA	NA
	Canto, 2006 [16]	EUS (ERCP, CT)	78	NR (3–12)	4	2	2	4/4, 100%	3/4, 75%	NA	NA
	Canto, 2012 [3]	EUS, MRCP, CT	216	29 (14–47)	2	0	2	2/2, 100%	2/2, 100%	1/2, 50%	1/2, 50%
	Canto, 2018 [17]	EUS, MRCP, CT	354	67 (NR)	24	14	10	NR	NR	NR	NR
	Dbouk, 2022 [18]	EUS, MRCP	1461	33 (0–67)	12	9	3	NR	NR	NR	NR
Marburg, Germany	Langer, 2009 [99]	EUS, MRCP	76	NR (0–60)	0	0	0	NA	NA	NA	NA
	Schneider, 2011 [100]	EUS, MRCP	72	44 (0–84)	2	1	1	NR	NR	NR	NR
	Bartsch, 2016 [101]	EUS, MRCP	253	28 (1-152)	6	2	4	NR	NR	NR	NR
	Bartsch, 2021 [82]	MRCP (EUS-FNA)	295	NR (NR)	5	2	3	NA	NA	5/5, 100%	3/5, 60%
Amsterdam / Rotterdam, Netherlands	Kluijt, 2009 [102]	EUS, MRI, CT	3	0	2	2	0	2/2, 100%	1/2, 50%	1/2,50%	0/2, 0%
	Poley, 2009 [103]	EUS (MRI, CT)	44	0	3	3	0	3/3, 100%	1/3, 33%	1/2,50%	0/2, 50%
	Harinck, 2015 [87]	EUS, MRCP	139	0	1	1	0	1/1, 100%	1/1, 100%	0/1, 0%	0/1, 0%
	Overbeek, 2022 [20]	EUS, MRCP	366	63 (0-156)	10	10	0	9/9, 100%	3/9, 33%	6/8, 75%	2/8, 25%
New York, USA	Verna, 2010 [104]	EUS, MRCP (ERCP)	51	0	2	2	0	2/2, 100%	1/2, 100%	2/2, 100%	1/2, 100%
Leiden, Netherlands	Potjer, 2013 [84]	MRCP (EUS)	241	35 (0-127)	12	9	3	NR	NR	NR	NR
	Vasen, 2016 [105]	MRCP, EUS (CT)	411	NR (0-169)	18	14	4	NR	NR	12/14 (86%)	5/14 (36%)
	Klatte, 2022 [19]	MRCP, EUS (CT)	347	67 (NR)	36	36	0	NR	NR	29/36 (81%)	12/36 (33%)
Toronto, Canada	Al-Sukhni, 2012 [106]	MRCP (EUS, CT, ERCP)	262	50 (0–96)	3	3	0	NA	NA	3/3 (100%)	0/3 (0%)
Milwaukee, USA	Sud, 2014 [107]	EUS (EUS-FNA)	16	0	2	2	0	3/3, 100%	1/3, 33%	NA	NA
Madrid, Spain	Mocci, 2015 [108]	EUS, CT (MRI)	38	NR (0–24)	1	0	1	1/1, 100%	1/1, 100%	NA	NA
Odense, Denmark	Joergensen, 2016 [109]	EUS	71	60 (2–92)	2	2	0	2/2, 100%	1/2, 50%	NA	NA
Houston, USA	DaVee, 2018 [110]	EUS, MRI, CT	86	30 (NR)	0	0	0	NA	NA	NA	NA
Haifa, Israel	Lachter, 2018 [111]	EUS	123	NR (NR)	1	1	0	1/1, 100%	0/1, 0%	NA	NA
Tampa, USA	Gangi, 2018 [112]	EUS	58	NR (0-120)	0	0	0	NA	NA	NA	NA
	McNamara, 2019 [113]	EUS	83	(0-120)	2	2	0	2/2, 100%	0/2, 0%	NA	NA
Verona, Italy	Paiella, 2018 [114]	MRCP, EUS	187	0	5	5	0	5/5, 100%	2/5, 40%	1/1, 100%	1/1, 100%
	Paiella, 2023 [85]	MRCP, EUS	156	NR (36-NR)	9	8	1	NR	NR	NR	NR
Liverpool, UK	Sheel, 2019 [83]	CT, EUS, MRI	321	24 (0–60)	1	1	0	NR	NR	NA	NA
New York, USA	Bar-Mashiah, 2020 [115]	EUS, MRCP	75	31 (2–73)	2	1	1	NR	NR	NR	NR
White Plains, USA	Raff, 2022 [116]	EUS, MRI	102	40 (NR)	0	0	0	NA	NA	NA	NA
Boston, USA	Shah, 2023 [86]	EUS, MRCP	252	NR (NR)	2	2	0	NR	NR	NR	NR
Barcelona, Spain	Llach, 2023 [117]	EUS, MRI	78	66 (NR)	1	1	0	1/1, 100%	0/1, 0%	NA	NA

*Abbreviations*: CT, computed tomography, ERCP, endoscopic retrograde cholangiopancreatography; EUS, endoscopic ultrasound; FNA, fine-needle aspiration; HGD, intraductal papillary mucinous neoplasm with high-grade dysplasia or pancreatic intraepithelial neoplasia-3; MRCP, magnetic resonance cholangiopancreatography; MRI, magnetic resonance imaging; NA, not applicable; NR, not reported; PDAC, pancreatic ductal adenocarcinoma

## Data Availability

No datasets were generated or analysed during the current study.
